# Then and Now

**Published:** 1897-05

**Authors:** 


### Editorial.

### THEN AND NOW.

Those whose experience in dentistry can antedate the last half of the century will remember the crudities in work at that time in many directions, but more especially in that of supplies. The assembling of articles needed in dental work under one roof was not then thought of any more than, if as much as, it is considered necessary to-day in general medicine and collateral surgical branches. The instrument-maker was then an important factor, and was supposed to be equal to the manufacturing of all necessary tools for excavating, filling, or extracting. The silversmith would prepare the solder and roll out the silver and gold when these could not be made in the laboratory. The aid of the machinist was often required for a special instrument, and even the teeth for artificial dentures, both before and after the advent of Stockton, were, in this country, carved and burned in private laboratories.

The change from this in fifty years has been so gradual that very few, it is imagined, ever stop in the busy whirl of every-day work to think of the marked difference between then and now.

From that period, beginning in the thirties, has been developed about all we know of modern dentistry in its several relations. The mind that fails to be impressed with this growth must be dull indeed and incapable of comprehending its significance in all its details.

It is feared that many in our profession have such an exalted idea of what is called its scientific side that they are incapable of observing with judicial accuracy that progress in any direction is not the result of one form of thought or practice, but must be attained through an aggregation of mind leading through diverse channels to the same end; and cannot be accomplished through a narrow-minded or contracted experience.

It is not uncommon to hear expressions like this: "Dentistry is a profession, not a trade," as though it were necessary to exalt the one at the expense of the other. The "mere mechanic" is a glib phrase too often heard, and that with disgust, by those who recognize in the mechanic of originality the highest type of advanced thought. The writer always stands before a complicated piece of mechanism with a certain feeling of awe and veneration as though in the presence of the nearest representative on earth among men of the Divine creative thought of the universe.

The lines should unquestionably be sharply drawn between professional work and that styled mechanical or commercial, but he who is unwilling to give due meed of praise to both the latter, as equal factors in progress, is incapable of reading history correctly.

The condition of things, in dental work, at the period named have all changed. The old Stockton teeth, massed by thousands in tin boxes to be laboriously selected, have given place to artistic arrangements on cards, in which some regard is had to natural appearances. Instead of the dentist running round to this shop and the other mechanic for tools, he finds them assembled to his needs under one roof. Instead of patronizing the silversmith for his silver or gold, he has these ready prepared for him by metallurgical experts; and instead of depending on hand-made instruments, no two alike, he expects to receive the finest steel made into instruments by machines constructed with mathematical accuracy, and always doing the same thing in the same way and with a perfection impossible of attainment by hand-labor.

With a view of coming into direct personal contact with the non-professional side of dentistry, the writer accepted the very kind invitation of Mr. W. H. Gilbert, general manager of the S. S. White Dental Manufacturing Company, to visit their works on Staten Island, New York. The reasons that influenced this visit were many, but perhaps the most important of all was the desire to compare the work of the present with that quite familiar in the long ago. That this desire was fully satisfied needs no assertion here. It was one of those days that come infrequently in men's lives in which, apparently, the flood-gates of intelligence are opened and the mind stands, as it were, oppressed by the weight of its own ignorance.

The dentists of this country who gather at the annual convocations and look upon the beautiful collections of exhibits scarcely give a thought to the amount of mental force and patient skill involved in their manufacture. They are to them simply instruments, the product of the mechanic. When, then, these unequally trained minds are brought, for the first time, in direct contact with the processes and mechanism necessary to produce these, they become simply appalled at their inability to grasp the harnessed ideas spread out for their contemplation.

It was with something of this feeling that the writer undertook, under the guidance of Mr. Gilbert and Mr. Johnston, the survey of about four acres of buildings with their contents. It requires a day's hard work to give merely a glance at the various processes, and a study of weeks, perhaps months, would be necessary to give a detailed description. It is, therefore, not the purpose of the writer to do this, but rather to give impressions of certain things.

It is, probably, the general thought that an instrument requires a certain degree of exactness in manufacture, but that it is necessary to bring this perfection to mathematical precision is, ordinarily, not a part of the dental comprehension. It is recognized in a general way that a watch can be made better by machinery than by hand, for that is of daily experience, but that the same absolute exactness of manufacture can be secured in dental appliances is, it is presumed, not generally considered or appreciated.

The first impression received is of the almost perfect unity and interdependence of each department of these great works with the other, and how by a wonderful system of organization errors are reduced to a minimum.

Another thought was uppermost in the mind during this examination, and perhaps more important than any other, that the mechanical side of dentistry is of as much value as the theoretical, and has been an equally pronounced factor in its advancement. When it is considered that this aggregation is but a part of the work of this house, and that it is but a fraction of a greater whole, distributed throughout the world, and that all this is the outcome of less than fifty years of effort, the question naturally arises, If this be the result of combined labor in that period, what may be expected from the first half of the coming century?

It is impossible to carry our readers from department to department, but to the dentist the metallurgical branch is full of interest, and to this we were first conducted. Here all the precious metals are refined and prepared for use. Here the sheets of gold are seen passing between powerful rollers so true that not a variation of a line is observed as they pass drawn down to the thousandths of an inch. There, glowing with intense heat too brilliant for the unprotected eye, was the platinum furnace, melting several thousand dollars of this increasingly valuable metal, which, in a few minutes flowed out into the mould with an intensity of light equal to the sun at mid-day, and equally unbearable. This is cooled with difficulty, and the writer handled, while still warm, the largest amount of platinum ever seen by him.

From here to the manufacture of burs. It is presumed the average dentist, if he thinks at all of machine-made burs, has the idea that these are prepared in blanks, then run into a machine, and come out burs as we have them. If any one has such an idea, the long rows of machines, each with an attendant, would dispel this crude conception. Each instrument is made with all the delicacy of those used in watch manufacture. Each has a certain part to perform, and from the beginning until the final test, the work is a combination of mental qualities with mechanical perfection. One of the most impressive sights to the writer was the machine which at that time was polishing the blades cut in one of the smallest burs made. The accuracy and precision with which this instrument turned the bur to meet the travelling polisher was marvellous, and sufficient to rouse the dullest intellect to a comprehension of the exactness to which all these processes had been brought.

Perhaps one of the most instructive sights, as it indicated the great care exercised, was the row of workmen with microscopes, the stage illumined by an incandescent light, examining each bur as it came from the machine. Any imperfection observed caused its condemnation. Whether machine-made burs are superior to hand-made is a question to be left to those who use them, but there can be no dispute with the statement that these operations are conducted with a precision not obtainable by any hand-work, however skilful it may be. When it is remembered that this result has been attained through the mechanical genius of one man, Mr. A. W. Browne, and twelve years of patient testing, a limited idea may be secured of what it cost to form even so small an instrument as a bur, where the highest standard of excellence is demanded. These machines once seen at work cause words to seem weak in comparison to the results achieved by this wonderful mechanical intellect.

Leaving the manufacture of burs reluctantly, we enter the place where all the tools are made that enter into the production of everything in this establishment. This cannot be described in this article, and it will be sufficient to say that this is not only extensive, but of vital importance, as the tools must be rigidly exact and as near perfect as the skilled mechanician can make them.

Through the various branches, each distinct in itself, we suddenly are ushered into the presence of huge gasometers, reminding one of the gas-works of a small country town. These contain oxygen and nitrous oxide, and from these, through a system of pipes, the contents are conducted to another department, and there stored in iron cylinders familiar to the profession. The operation of filling these is of much more interest than that usually made familiar over the office chair.

The perfect organization of this extensive plant, where, of necessity, everything must have its place and be kept there always prepared for immediate use, is strikingly manifested in the arrangement of the tools. These are all placed in proper receptacles, the small upon prepared boards of regulation size for sliding in drawers and the larger instruments in separate cases. Each has its proper number and the purpose for which it is to be used carefully marked upon it, and then registered in a book kept for the purpose. When the workman wishes to prepare a certain portion of a hand-piece, as an illustration, the tools necessary must be applied for and a receipt given. This insures care upon the part of the workman and a certainty that all are in readiness to complete orders. The capital locked up in these innumerable drawers and cases can hardly be estimated.

The distinct parts of every appliance manufactured here must come together to form the perfect instrument, almost without the use of a file, and so exact is this that the separate pieces of the chairs are sent from the shops to the japanning-room with the certainty that they will go together without future injury to the surface.

When it is remembered that these separate parts must be made in vast quantities and stored ready to be put together at short notice, one is not surprised to find rooms of large size filled with stacks of various forms awaiting the assembling and final finish.

A hasty glance through the various departments devoted to the manufacture of rubber materials used in dentistry; the preparation of modelling compound and the various forms of wax; the interesting operation of twisting the cable wires for the engine; the weaving of the cords to run these and also the covers for rubber tubing, furnish a fitting close to the day's inspection.

Much might be said of the Johnston Brothers in this connection. Their scientific ability, combined with a broad collegiate culture of the old New England type, has abundantly fitted them for the immediate management of this intricate and responsible work; part only, it is true, but a very important part of the S. S. White Dental Manufacturing Company's system.

To some it may seem as though this article was out of place in a dental journal devoted to professional topics, but the writer views it quite otherwise. From his boyhood he has intimately mingled with the mechanical side of dentistry, and has followed the fortunes of this house from its earliest inception, and has learned throughout it all that advancement in any line of work must be through many and oftentimes, apparently, diverse avenues, but, in the end, they meet upon one common level. Dentistry without its mechanical companion would be helpless, and the mechanical work without its complement, the professional, would cease to exist. It is, therefore, held that while the dental profession is not in full agreement with all the methods of trade, and can never be, it should recognize that where a firm wastes immense sums yearly to bring the tools and materials used in dentistry to perfection, and is satisfied with comparatively meagre returns as a profit on capital, selfishness cannot be charged as one of the over-mastering sins of all trade-workers. In our criticism let us learn to be just, and at the same time be sure that we also have passed through the furnace of a world's selfishness and have garments upon which there is no odor of fire.

